# Direct Growth of 2D Bilayers on Au(111) for Low‐Coercive Sliding Ferroelectricity

**DOI:** 10.1002/adma.74537

**Published:** 2026-08-05

**Authors:** Honglin Chen, Ke Yang, Yuhuan Li, Lingli Huang, Tianren Chen, Ming Yang, Jiong Zhao, Thuc Hue Ly

**Affiliations:** ^1^ Department of Chemistry and Center of Super‐Diamond & Advanced Films City University of Hong Kong Kowloon China; ^2^ Department of Physics and Materials The Hong Kong Polytechnic University Kowloon China; ^3^ City University of Hong Kong Shenzhen Research Institute Shenzhen China; ^4^ Research Centre on Data Sciences & Artificial Intelligence The Hong Kong Polytechnic University Kowloon China; ^5^ Research Centre for Nanoscience and Nanotechnology The Hong Kong Polytechnic University Kowloon China; ^6^ The Hong Kong Polytechnic University Shenzhen Research Institute Shenzhen China; ^7^ The Research Institute for Advanced Manufacturing The Hong Kong Polytechnic University Kowloon China; ^8^ Department of Chemistry and State Key Laboratory of Marine Environmental Health City University of Hong Kong Kowloon China; ^9^ Hong Kong Institute for Clean Energy City University of Hong Kong Kowloon China

**Keywords:** bilayer ReS_2_, CVD growth, sliding ferroelectricity, stacking order

## Abstract

Two‐dimensional (2D) semiconductors directly grown on metal substrates can enable pristine and low‐defect interfaces that are difficult to achieve through transfer‐based fabrication. Although monolayer transition metal dichalcogenides (TMDs) have been widely synthesized on metals, controllable bilayer growth remains challenging owing to distinct thermodynamics and kinetics of second‐layer growth on inert first‐layer surfaces—a significant hurdle given their importance for interlayer‐coupled functionalities like sliding ferroelectricity. Low‐symmetry rhenium dichalcogenides (ReX_2_, X = S, Se) are especially attractive because their weak interlayer coupling and multiple thermodynamically stable stacking configurations support low sliding barriers. Here, by optimizing precursor flux and growth kinetics, we achieved controlled chemical vapor deposition (CVD) growth of bilayer ReS_2_ with well‐defined parallel and antiparallel stacking directly on Au(111). As‐grown parallel‐stacked bilayers exhibit a large piezoelectric response (effective *d*_33, eff_ = 9.84 pm V^−1^) and sliding ferroelectricity with an ultralow coercive voltage of approximately 2 V. Tip‐defined ferroelectric tunnel junction and ferroelectric field‐effect transistor measurements further support the ferroelectric interpretation. Comparative studies of as‐grown, transferred, and exfoliated bilayers reveal that the cleaner, more conformal interface of the as‐grown bilayers is associated with a lower coercive bias. This work establishes a scalable route for stacking‐specific bilayer TMD growth on metal toward low‐coercive sliding ferroelectricity.

## Graphical abstract

Controlled CVD growth of stacking‐defined bilayer ReS_2_ on Au(111) enables parallel‐stacked bilayers with intrinsic sliding ferroelectricity, a strong piezoelectric response, and an ultralow coercive voltage. Atomic‐resolution imaging and in situ measurements further reveal the decisive role of clean interfaces in achieving low‐barrier ferroelectric switching in low‐symmetry bilayer ferroelectric systems.

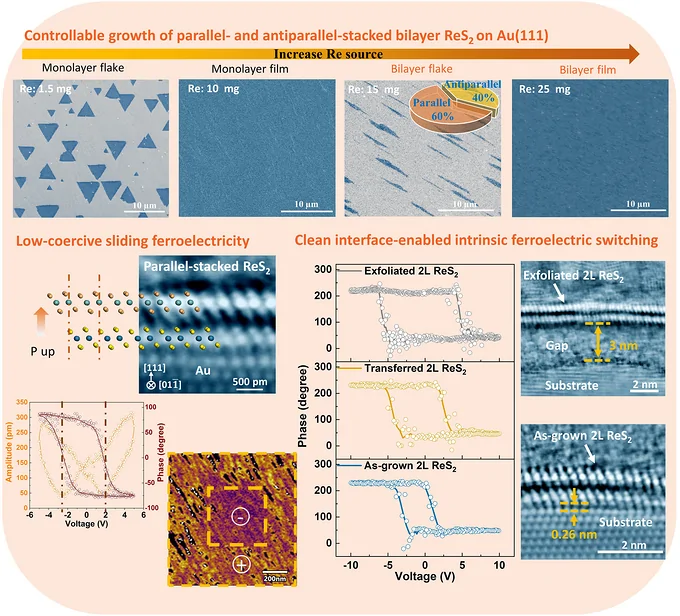

## 1 Introduction

Two‐dimensional (2D) semiconductors, such as transition metal dichalcogenides (TMDs) including MoS_2_, WSe_2_, ReS_2_, have provided a versatile platform for next‐generation electronics [1, 2, 3, 4]. Chemical vapor deposition (CVD) remains the most scalable method for synthesizing 2D transition metal dichalcogenides (TMDs). Indeed, wafer‐scale growth of monolayer TMDs is now well‐established, particularly on insulator substrates such as SiO_2_/Si, sapphire, mica, and glasses [5, 6, 7, 8]. However, sophisticated device integration necessitates the direct CVD growth of 2D semiconductors onto metal substrates. Direct growth of 2D semiconductors on metal yields significantly cleaner metal‐2D semiconductor interfaces [9, 10, 11] compared to post‐growth transfer methods [12, 13, 14] and offers superior structural quality over post‐growth metal deposition for electrode fabrication. Despite these advantages, the controllable growth of 2D TMDs on metal surfaces remains challenging, primarily due to the excessive catalytic activity of the metals and their high precursor adsorption capacity, which can introduce complex kinetic pathways that are difficult to regulate during synthesis.

While monolayer TMDs growth on metals has been widely achieved via CVD methods [11, 15, 16], achieving 2D bilayer growth is more challenging on metal surfaces because of the starkly different surface energies of metals and 2D layers. Specifically, the second layer must nucleate on the inert van der Waals (vdW) surface of the first layer, where precursor capture is significantly reduced and the nucleation barrier is much higher [17]. Besides, achieving bilayer formation without triggering uncontrolled multilayer stacking requires overcoming the bilayer nucleation barrier within a narrow kinetic window defined by precursor flux and growth conditions [18]. The mismatched reaction activities, precursor capture capabilities, and surface diffusion rates between the metal and the initial TMD layer make the formation of uniform bilayers with controlled stacking configurations particularly difficult. Consequently, the direct growth of bilayer TMDs with controlled thickness and well‐resolved stacking configurations on metal surfaces remains substantially less developed than monolayer growth.

Meanwhile, 2D bilayers exhibit unique functionalities emerging from interlayer coupling [19, 20, 21, 22, 23, 24], most notably sliding ferroelectricity [25, 26, 27]. Unlike conventional ionic‐displacement ferroelectrics, sliding ferroelectricity arises from interlayer registry shifts that break inversion symmetry, enabling polarization switching without bond breaking and potentially lowering the switching barrier [28, 29, 30]. Although demonstrated in several bilayer systems, including hexagonal boron nitride (hBN) [25], MoS_2_ [31, 32, 33, 34], WTe_2_ [26], and MoS_2_/WS_2_ [35], achieving low‐coercive ferroelectric switching in device‐relevant configurations still requires further efforts. A key limitation lies not only in the material itself, but also in the device configuration and interface quality. In practical vertical ferroelectric devices, such as ferroelectric tunnel junctions (FTJs), efficient polarization switching requires well‐defined metal‐ferroelectric interfaces to ensure effective electric field application. However, most bilayer 2D ferroelectric systems are currently fabricated through post‐growth electrode deposition or mechanical exfoliation and transfer [36, 37, 38]. These processes inevitably degrade interface integrity, introduce structural damage, and result in unwanted interfacial gaps or contaminants [12, 13, 14, 39]. These act as parasitic dielectric barriers, causing a significant voltage drop outside the ferroelectric layer, elevating the switching barrier and degrading device performance [40, 41, 42].

In addition to synthesis and device fabrication challenges, the choice of material system is crucial for realizing low‐barrier sliding ferroelectricity in bilayers. Most previous studies have focused on high‐symmetry materials, where relatively strong interlayer coupling and limited stacking configurations restrict the accessible polarization states [25, 26, 31]. In contrast, low‐symmetry 2D materials with weak interlayer interaction, for example, rhenium dichalcogenides (ReX_2_, X = S, Se), have demonstrated a richer variety of thermodynamically favorable bilayer slip stacking (parallel and antiparallel stacking), and have low energy barrier between these stacking [43], making them ideal candidates for intrinsically low‐coercive sliding ferroelectricity. Previous studies have reported out‐of‐plane ferroelectricity in mechanically exfoliated antiparallel‐stacked bilayer ReX_2_ and sliding‐induced polarization in parallel‐stacked multilayer ReSe_2_ [44, 45, 46].

Here, we demonstrate the CVD synthesis of bilayer ReS_2_ with well‐defined parallel and antiparallel stacking directly on Au(111) substrates, forming a clean interface compatible with vertical device architectures. During the growth, the Au(111) substrate initially aligns the first ReS_2_ layer (details in our prior study [11]), which later serves as a template for the growth of the second layer, enabling it to inherit the same Re‐chain orientation and thus realize bilayer parallel or antiparallel stacking under optimized growth conditions. Piezoresponse force microscopy (PFM) reveals a large out‐of‐plane piezoelectric response and intrinsic sliding ferroelectricity in parallel‐stacked bilayer ReS_2_, characterized by an ultralow coercive voltage of approximately 2 V measured directly on the growth substrate. The real atomic structures for polar states are resolved by cross‐sectional scanning transmission electron microscopy (STEM) and consistent with the results from first‐principles calculations. Furthermore, systematic comparisons between as‐grown, transferred, and exfoliated parallel‐stacked bilayer ReS_2_ reveal a strong correlation between sample configuration, interfacial quality, and the measured ferroelectric response, highlighting the advantage of direct growth in minimizing transfer‐related interfacial artifacts. This work demonstrates the scalable CVD synthesis of parallel‐ and antiparallel‐stacked bilayer ReS_2_ on gold and provides insights into intrinsic low‐coercive ferroelectricity in the parallel‐stacked configuration, underscoring the importance of direct growth on metals for next‐generation electronic applications.

## 2 Results and Discussion

### 2.1 Controllable Bilayer ReS_2_ Growth on Gold Substrate

In this study, large‐area Au(111) substrates were first prepared following our previous established annealing method [11], and a representative electron backscatter diffraction (EBSD) data confirmed the formation of Au(111) domain with centimeter scale (Figure 1a). The sulfur powder and ammonium perrhenate (NH_4_ReO_4_) were adopted as the S and Re sources, respectively. Bilayer ReS_2_ was then synthesized via CVD with a double‐zone furnace (Figure 1b). ReS_2_ is a low‐symmetry group‐VII transition‐metal dichalcogenide that adopts a distorted 1T structure. The in‐plane structural distortion causes the Re atoms to form diamond‐shaped Re_4_ units connected along the Re‐chain direction, resulting in pronounced structural anisotropy. The conventional crystal structure of ReS_2_ is described by the triclinic space group P‐1. Importantly, this layer symmetry alone does not uniquely determine the overall symmetry of a bilayer, which also depends on the relative orientation and lateral registry of the two constituent layers, as further discussed in Sections 2.2 and 2.3.

**FIGURE 1:**
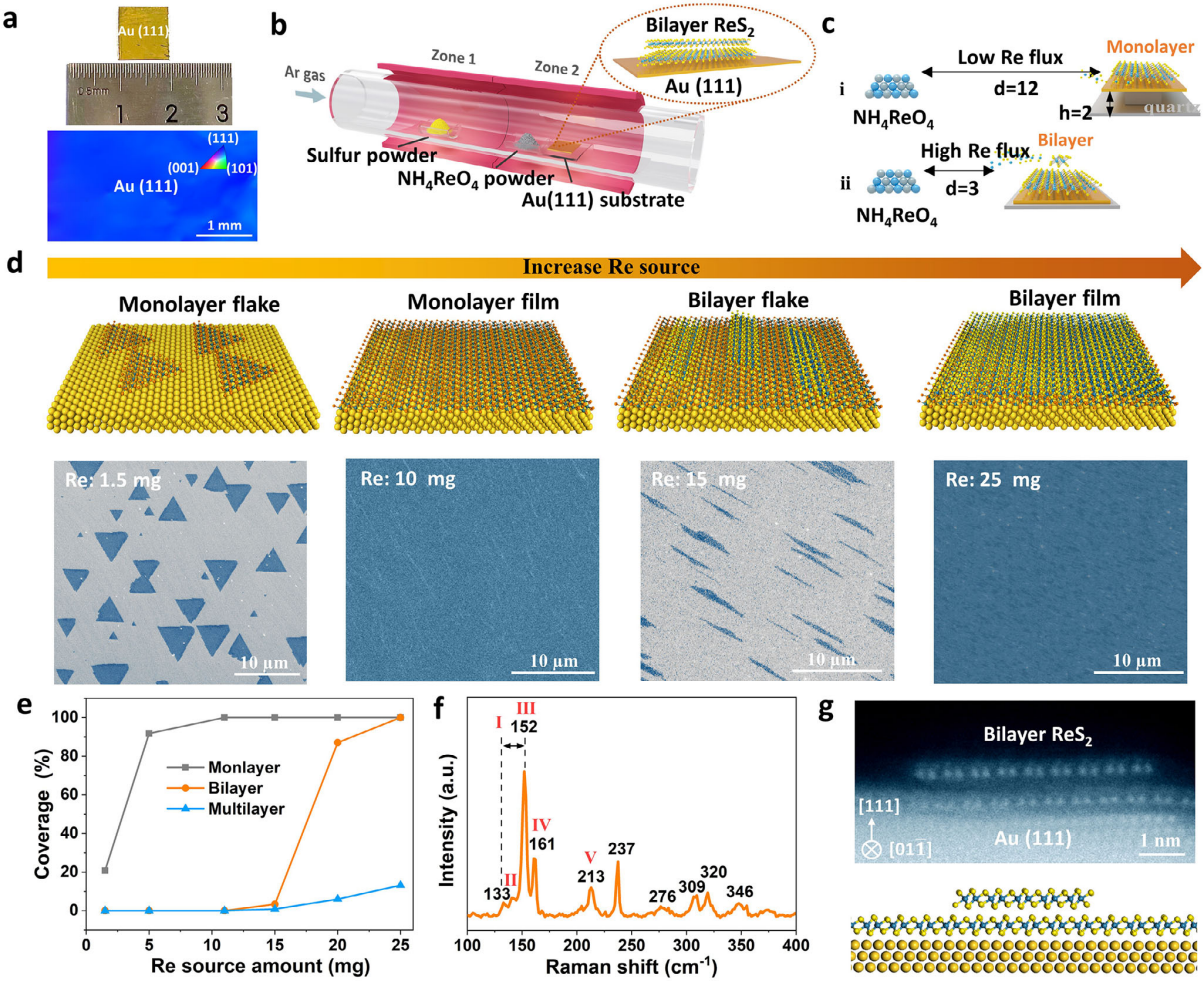
CVD growth of bilayer ReS_2_ on Au(111). (a) Optical image of obtained centimeter‐size Au(111) after annealing and corresponding EBSD map. (b) Schematic illustration of the CVD setup for bilayer ReS_2_ growth. (c) Schematic of the CVD environment under (i) low and (ii) high Re flux conditions. (d) Schematic and false‐color SEM images showing the growth evolution from monolayer to bilayer ReS_2_ with increasing Re source amount. (e) The coverage plots of monolayer, bilayer, and multilayer ReS_2_ as a function of Re source amount. (f) Raman spectrum of bilayer ReS_2_ grown on Au(111). (g) False‐color cross‐sectional HAADF‐STEM image and corresponding atomic model of a bilayer ReS_2_ flake on Au(111).

To elucidate the parameters governing bilayer ReS_2_ synthesis, we systematically investigated the influence of various growth conditions. Our previous setup with a long Re–substrate distance (Figure 1c, i), optimized for monolayer growth, utilized a high temperature (900°C–950°C) and a specific Re precursor amount. Building upon this configuration, we initially attempted to grow bilayers by using smaller Au foils and further increased precursor quantities. However, these efforts still yielded only continuous monolayer films (Figure S1), indicative of a “self‐limiting”‐like growth behavior analogous to graphene growth on Cu [47, 48]. We attribute this failure to a kinetically limited, “Re‐poor” environment caused by the long vapor transport pathway, where the precursor flux is insufficient to overcome the energy barrier for second‐layer nucleation on the van der Waals surface of the first layer.

Subsequently, we optimized the key parameters, including the source‐substrate distance (*d*), growth temperature (Figure S2), and substrate height (*h*) within the furnace (Figure S3). The optimal configuration for bilayer growth was identified as *d* = 3 cm, *T* = 800°C–850°C, and *h* = 0 cm (Figure 1c, ii). Among these, reducing the Re‐substrate distance is paramount. The shortened distance ensures a high precursor flux and local supersaturation at the substrate surface, effectively switching off the “self‐limiting” monolayer growth and making the nucleation and growth of the second layer thermodynamically favorable and kinetically feasible. Specifically, the enhanced Re flux increases the local supersaturation at the ReS_2_/Au(111) surface, which effectively compensates for the surface‐energy penalty associated with creating a new top ReS_2_ layer. Consequently, the net thermodynamic driving force for second‐layer nucleation becomes less unfavorable, leading to a reduced Gibbs free‐energy barrier for bilayer formation. At the same time, the higher adatom impingement rate kinetically promotes the formation of stable nuclei by increasing the probability that adatoms aggregate into a critical nucleus before they desorb. Notably, at this short distance, the original high growth temperature (900°C–950°C) led to the formation of byproducts, as evidenced by anomalous Raman peaks (Figure S2). A moderately lower temperature (800‐850°C) strikes a critical balance, suppressing parasitic reactions while maintaining sufficient adatom mobility for epitaxial bilayer growth.

Using this optimized setup, by gradually increasing the amount of Re precursor, we sequentially achieved monolayer flakes, continuous monolayer films, bilayer flakes, and finally, extensive bilayer films (Figure 1d). The growth follows a strict layer‐by‐layer mode, with no bilayer nucleation occurring prior to full monolayer coverage. The ReS_2_ with pure bilayer coverage remains challenging due to the concurrent nucleation of multilayers. We statistically quantified the coverage of monolayer, bilayer, and multilayer regions as a function of precursor amount (Figure 1e; Figure S4). Bilayer flakes begin to emerge after complete monolayer coverage is attained at a precursor mass of approximately 15 mg, with coverages of 3.3% (bilayer) and 0.8% (multilayer). At approximately 20 mg, the bilayer coverage increases dramatically to 87%, while the multilayer coverage rises to 6%. A further increase to approximately 25 mg yields complete bilayer film coverage (100%) with a multilayer proportion of ∼13.2%. This suggests a layer‐by‐layer growth mode, where a critical supersaturation threshold must be reached to enter the kinetic regime necessary for the formation of upper‐layer nuclei. Consistent with the coverage statistics, Raman mapping over a 50 × 50 µm^2^ area shows a relatively uniform intensity of the characteristic Raman mode at peak 213 cm^−1^ across most of the film, with only a few higher‐intensity regions corresponding to multilayer islands (Figure S5). This result further supports the large‐area thickness uniformity of the as‐grown bilayer film.

Besides, to suppress multilayer formation, we tried introducing hydrogen into the CVD process. While this effectively reduced the multilayer ratio, it came at the cost of a lower bilayer coverage and the formation of irregularly shaped flakes (Figure S6). This likely arises from hydrogen‐enhanced etching and reduced adatom residence time, which impede the formation of stable bilayer nuclei. In summary, the shortened vapor transport pathway and optimized precursor flux provide the critical conditions required to overcome the intrinsic nucleation barrier imposed by the weakly coupled ReS_2_ system.

Raman spectroscopy confirms the characteristic signals of bilayer ReS_2_ (Figure 1f). The frequency difference of approximately 19 cm^−1^ between Raman mode III (152 cm^−1^) and mode I (133 cm^−1^) indicates an AB‐stacked bilayer configuration according to previous report [49]. Furthermore, angle‐resolved polarized Raman (ARPR) was also employed to characterize bilayer ReS_2_, in which maximum intensity polarization direction of mode III is rotated clockwise with respect to that of mode V (Figure S7a), demonstrating the orientation of bilayer ReS_2_ follows $\bar{c}$‐up structure (Figure S7b) [50, 51, 52]. While Raman spectroscopy provides macroscopic structural information, the specific atomic stacking order, determined by subtle interlayer sliding, requires higher‐resolution techniques.

To unequivocally determine the stacking configuration of bilayer ReS_2_ grown on gold, cross‐sectional samples were prepared via focus ion beam (FIB) and analyzed using high‐angle annular dark‐field scanning transmission electron microscope (HAADF‐STEM). On the preparation of the cross‐sectional sample, the FIB cut direction was perpendicular to the long edge of ReS_2_ (Re‐chain direction) of bilayer flake, and STEM images were acquired along Au [$01\bar{1}$] zone axis. The result (Figure 1g) shows that both up and bottom ReS_2_ layers exhibit identical side‐view projections of Re atoms, indicating that they share the same Re‐chain direction aligned with the Au [$01\bar{1}$] direction, consistent with our previous observations in monolayer ReS_2_ growth.

### 2.2 Epitaxial and Stacking Order Analysis of Bilayer ReS_2_

The identical Re‐chain orientation observed in both ReS_2_ layers suggests an epitaxial inheritance growth behavior during bilayer formation on Au(111). In our prior study [11], we demonstrated that monolayer ReS_2_ growth is guided by the step edges of the Au(111) substrate, resulting in a well‐aligned monolayer film. This aligned first layer here serves as a structural template for growth of the second layer, allowing it to naturally inherit the same Re‐chain (b‐axis) direction along Au [$01\bar{1}$] direction. A schematic illustration of the epitaxial relationship between bilayer ReS_2_ and Au(111) is shown in Figure 2a. To further verify this growth behavior, bilayer ReS_2_ grown on Au(111) was transferred onto TEM grids for top‐view STEM characterization. High‐resolution HAADF‐STEM images reveal that the two ReS_2_ layers preserve the same Re‐chain orientation, as evidenced by the identical Re atomic arrangements in both layers (Figure 2b), despite the presence of diverse stacking configurations (Figure S8a–f) arising from interlayer sliding. Consistently, selected‐area electron diffraction (SAED) patterns collected from multiple bilayer samples exhibit only a single set of diffraction spots (Figure S8g–i), confirming the absence of interlayer rotational mismatch.

**FIGURE 2:**
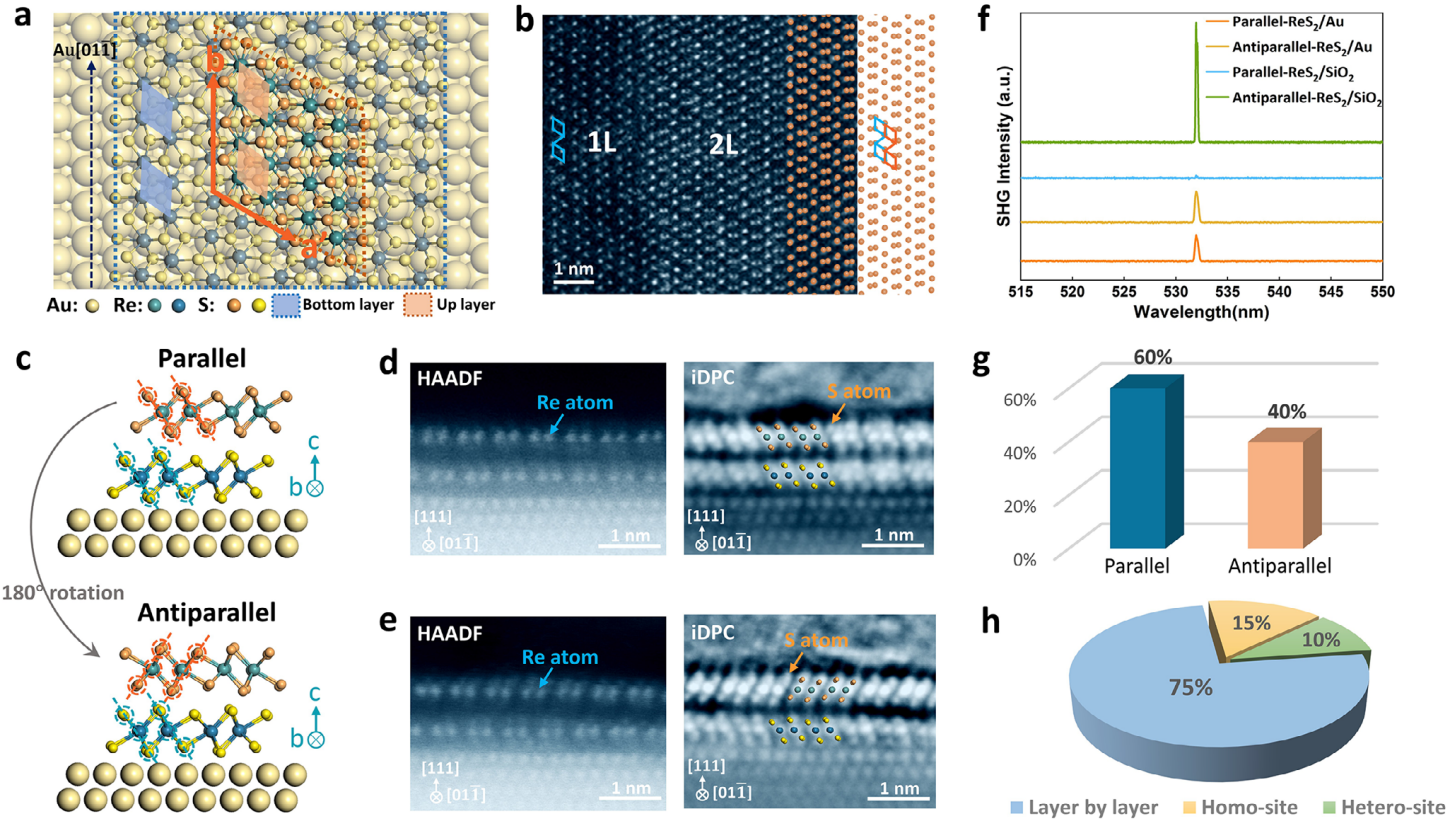
Interlayer stacking configurations of bilayer ReS_2_ grown on Au(111). (a) Top‐view atomic model illustrating the epitaxial relationship between bilayer ReS_2_ and Au(111). (b) High‐resolution top‐view HAADF‐STEM image of a representative bilayer ReS_2_, showing identical Re‐chain orientations in the two layers. (c) Side‐view atomic models of parallel‐ and antiparallel‐stacked bilayer ReS_2_ on Au(111). (d,e) Cross‐sectional HAADF‐ and corresponding iDPC‐STEM images of parallel‐ and antiparallel‐stacked bilayer ReS_2_ grown on Au(111), respectively. (f) SHG responses of parallel‐ and antiparallel‐stacked bilayer ReS_2_ measured on Au and SiO_2_/Si substrates. (g) Statistical distribution of parallel and antiparallel stacking in bilayer ReS_2_. (h) Statistical analysis of the nucleation modes of bilayer ReS_2_ growth.

To further resolve the detailed interlayer stacking configurations of bilayer ReS_2_ grown on Au(111), cross‐sectional imaging was subsequently performed using integrated differential phase‐contrast STEM (iDPC‐STEM), which provides enhanced sensitivity to light elements such as sulfur. Multiple stacking configurations (Figure S9) were identified and can be classified into two primary categories: parallel stacking and antiparallel stacking (Figure 2c). These two configurations are related by a 180° in‐plane rotation of the top layer. Although their top‐view projections appear nearly identical due to the intrinsic 180° rotational symmetry of the Re‐chain parallelogram unit, the side‐view structures exhibit distinct sulfur atomic arrangements. Notably, “parallel” and “antiparallel” describe the relative in‐plane crystallographic orientation of the two ReS_2_ layers rather than a unique interlayer atomic registry. Relative in‐plane translations between the two layers can generate different interlayer registries, and the symmetry of the bilayer depends on the specific atomic configuration. Figure 2d,e show representative cross‐sectional STEM images of parallel‐ and antiparallel‐stacked bilayer ReS_2_ grown on Au(111), respectively. The HAADF‐STEM images clearly resolve the Re atomic framework and confirm the high structural quality of the bilayers, while the corresponding iDPC‐STEM images provide enhanced sensitivity to sulfur atoms, enabling direct identification of parallel and antiparallel stacking through their different sulfur alignments. Overall, the above analyses clarify the epitaxial growth behavior and stacking configurations of bilayer ReS_2_ on the Au(111) surface.

It is worth noting that second‐harmonic generation (SHG) intensity can serve as an effective way to distinguish bilayer ReS_2_ stacking configurations. Specifically, the SHG signal from antiparallel‐stacked bilayers is approximately two orders of magnitude stronger than that of parallel‐stacked bilayers. This conclusion is validated by cross‐sectional STEM measurements on transferred bilayer samples exhibiting strong and weak SHG signals (Figure S10). Notably, this pronounced difference in SHG intensity between the two stacking configurations is not readily distinguishable when measured directly on the Au substrate (Figure 2f). This is primarily due to the strong surface SHG signal of gold [53] and its coherent superposition with the SHG signal from ReS_2_, which introduces a substantial background that masks the intrinsic differences between two stacking configurations. Moreover, the high reflectivity and optical loss of Au may reduce interference modulation compared to dielectric substrates such as SiO_2_/Si [54, 55], where multiple reflections enhance contrast. As a result, stacking‐dependent variations in the nonlinear susceptibility of ReS_2_ are difficult to resolve on Au but become clearly distinguishable after transfer to SiO_2_/Si substrates. Figure 2f clearly compares the SHG responses measured on SiO_2_/Si and Au substrates. Furthermore, based on SHG measurements of transferred samples, we statistically analyzed 105 bilayer flakes and quantified the distribution of stacking configurations. The results indicate that parallel‐stacked ReS_2_ is slightly more prevalent, accounting for approximately 60% (63 of 105) (Figure 2g).

In addition, we statistically analyzed 20 cross‐sectional bilayer ReS_2_/Au samples (Figure 2h; Figure S11) to clarify the nucleation behavior of bilayer ReS_2_. Among them, 75% (15/20) follow a layer‐by‐layer growth mode, in which the second ReS_2_ layer nucleates on top of an already‐grown first ReS_2_ layer. In contrast, 15% (3/20) and 10% (2/20) exhibit homo‐site and hetero‐site nucleation, respectively, where the second‐layer nucleus forms either at the same or at a different lateral position relative to the first‐layer nucleus before the underlying layer is fully developed. These results confirm that layer‐by‐layer growth is the dominant growth pathway for bilayer ReS_2_ on Au(111). Among 11 identified stacking orders in these 20 samples (Figure S11), 55% (6/11) is parallel stacking, agreeing well with the statistic result of Figure 2g.

### 2.3 Theoretical Analysis of Sliding Ferroelectricity of Parallel‐Stacked Bilayer ReS_2_

The presence of diverse stacking arrangements in bilayer ReS_2_ suggests the possibility of potential piezoelectric and sliding ferroelectric behavior. Density functional theory (DFT) calculations were subsequently performed to investigate the sliding ferroelectric behavior of both parallel‐ and antiparallel‐stacked bilayer ReS_2_ (Figure 3; Figure S12). During the calculations, the upper ReS_2_ layer was displaced along the a and b axes of the ReS_2_ crystal by fractional distances of the lattice constants (*a* = 0.65 nm and *b* = 0.64 nm) within one unit cell area, sampling 100 distinct stacking orders. The resulting energy landscape for these parallel‐stacked bilayers ReS_2_ is shown in Figure 3b. Two energetically favorable polar states with opposite polarization directions, denoted as polar states *A* and *B*, are identified at positions of (0.5a, 0b) and (0.3a, 0.5b), respectively. An intermediate nonpolar state *C* is located at (0.4a, 0.2b), corresponding to a relatively higher energy region. The atomic configurations of polar states *A* and *B* are depicted in Figure 3c, and the calculated spontaneous polarization values are 0.0035 pC/m (upward) and −0.0014 pC/m (downward), respectively. An ideal bulk‐like parallel‐stacked ReS_2_ structure composed of two equivalent layers retains inversion symmetry. However, parallel stacking specifies only the relative in‐plane orientation of the two layers and does not uniquely determine the symmetry of a specific interlayer registry. The nonzero calculated polarizations of states *A* and *B* indicate inversion‐symmetry breaking in these configurations.

**FIGURE 3:**
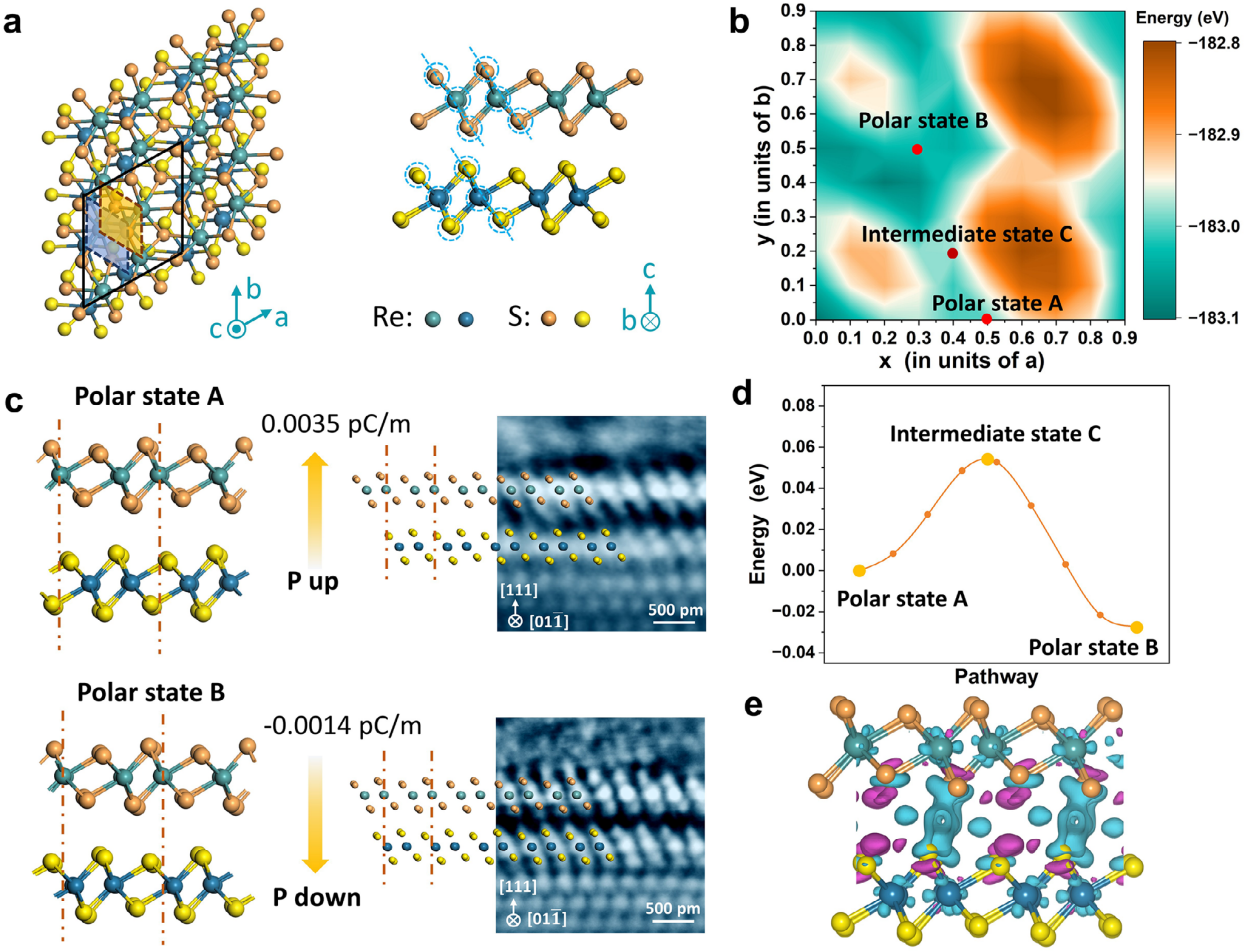
Theoretical analysis of sliding ferroelectricity in parallel‐stacked bilayer ReS_2_. (a) Top‐ and side‐view atomic models of parallel‐stacked bilayer ReS_2_. (b) The energy map of bilayer ReS_2_ as a function of sliding distance along the *a* and *b* axes. Two ferroelectric states (polar states *A* and *B*) and the intermediate state *C* are indicated by red dots. (c) The side‐view atomic models of polar states *A* and *B* and the corresponding iDPC‐STEM images. (d) The energy pathway for the ferroelectric transition from polar state *A* to *B*. (e) The charge density difference of bilayer ReS_2_ in polar state *A*. The purple and blue regions represent charge accumulation and depletion, the iso‐surface is 10^−4^ e/Å^3^_,_ respectively.

Importantly, the corresponding atomic structures of these polar states were directly observed in as‐grown bilayer ReS_2_ on Au(111) using iDPC‐STEM, providing experimental validation of the theoretical predictions. The estimated energy barrier (Figure 3d) for polarization switching by interlayer sliding, corresponding to the transition from polar state A to the intermediate state *C* (from polar state *B* to intermediate state *C*), is 0.054 eV (0.081 eV), which suggests the low coercivity in our polarization switching experiment by PFM. To elucidate the origin of the out‐of‐plane ferroelectric polarization, charge density difference calculations were performed for polar state *A*. As shown in Figure 3e, pronounced electron accumulation and depletion occur at the interlayer interface, accompanied by a net charge transfer from the top layer to the bottom layer, which gives rise to an upward polarization.

Equivalent DFT analyses for antiparallel‐stacked bilayer ReS_2_ are presented in Figure S12. Although a similar theoretical study on antiparallel‐stacked ReS_2_ has been reported previously [44], the calculated atomic configurations of the two polar states (A’ and B’) in our work are consistent with that earlier report. Notably, considering the vdW (D3) correction, our calculated spontaneous polarization value (∼0.43 pC/m; Figure S12c) is larger than previously reported calculation values without vdW correction (∼0.07 pC/m) [44]. More importantly, in contrast to the earlier study that lacked direct experimental correlation, we directly observed these two polar‐state atomic configurations in our as‐grown bilayer ReS_2_ on Au(111) (Figure S12c). This establishes a direct correlation between theoretical predictions and experimentally realized structures for antiparallel‐stacked bilayer ReS_2._ The observation is further supported by the calculated low energy barrier for polarization switching in the antiparallel‐stacked configuration, approximately 0.17 eV (Figure S12d), higher than the parallel‐stacked configuration above.

Furthermore, a comparison of the energy landscapes for parallel‐ (Figure 3b) and antiparallel‐stacked (Figure S12b) bilayer ReS_2_ reveals that the lowest‐energy configurations in both cases are nearly identical, with energies close to −183.08 eV. The numbers of stacking configurations (109 in total) within the low‐energy range (−183.1 to −183.0 eV) are also comparable, accounting for 57 out of 109 (≈52%) configurations for parallel stacking and 52 out of 109 (≈48%) for antiparallel stacking. This theoretical preference is also consistent with the statistical results obtained in the previous section from SHG measurements (≈60%, Figure 2g) and cross‐sectional STEM analysis (≈55%, Figure S10).

### 2.4 Piezoelectricity and Sliding Ferroelectricity of Parallel‐Stacked Bilayer ReS_2_

The predicted sliding ferroelectricity in parallel‐stacked bilayer ReS_2_ was further investigated experimentally. First, clear SHG responses have been observed from both as‐grown and transferred parallel‐stacked bilayer ReS_2_ on Au and SiO_2_/Si substrates in the previous section (Figure 2f), which already confirms its non‐centrosymmetric nature. We further did power‐dependent SHG measurements for parallel‐stacked bilayer ReS_2_ (Figure 4a), where the SHG intensity increases monotonically with increasing excitation power. The extracted SHG intensity exhibits a linear dependence in the log‐log plot with a slope of 1.85 (Figure S13), close to the theoretical value of 2 for a second‐order nonlinear optical process. These SHG results collectively demonstrate the broken inversion symmetry in as‐grown parallel‐stacked bilayer ReS_2_.

**FIGURE 4:**
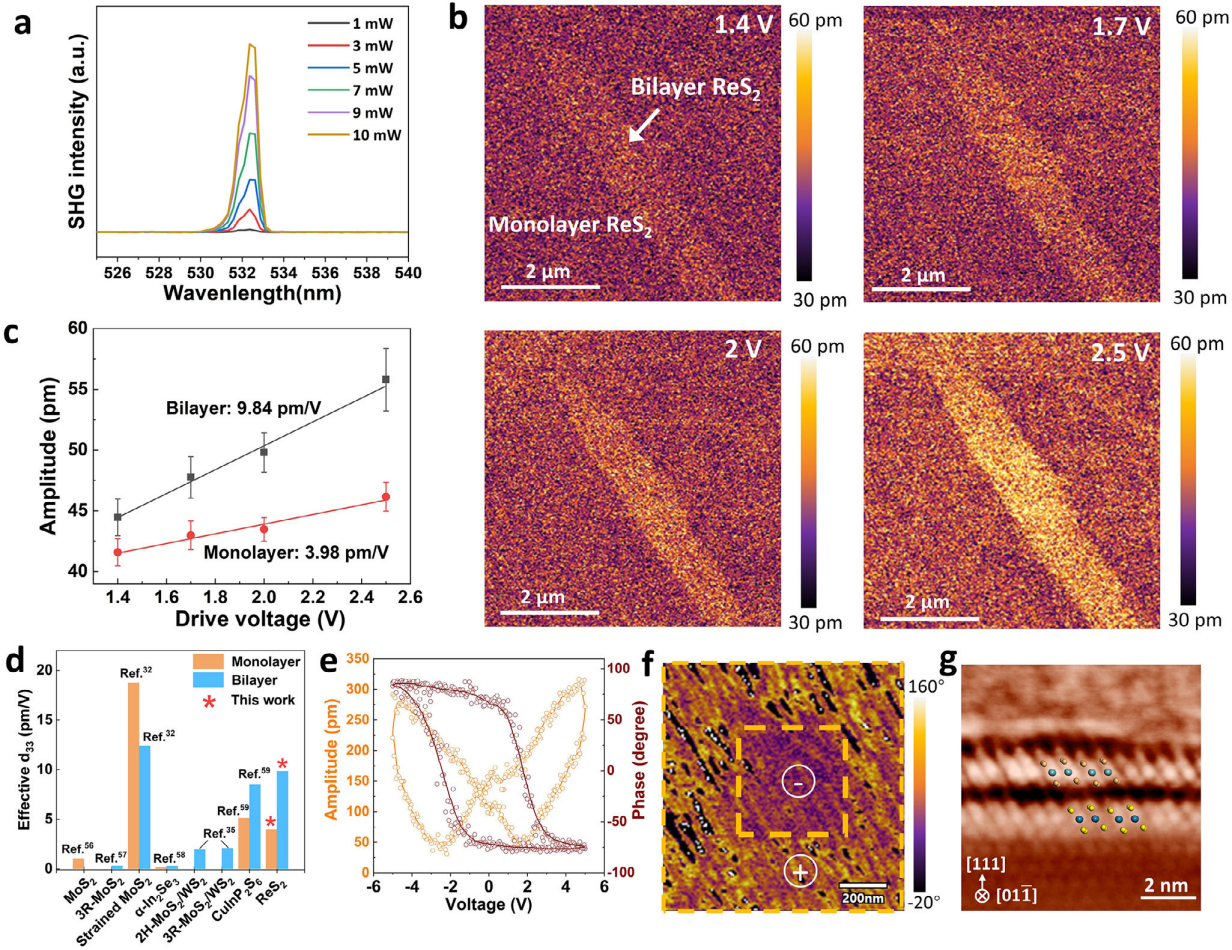
Piezoelectricity and sliding ferroelectricity characterization of parallel‐stacked bilayer ReS_2_ grown on Au(111). (a) SHG spectra of parallel‐stacked bilayer ReS_2_ measured under increasing excitation power. (b) PFM amplitude images of the parallel‐stacked bilayer ReS_2_/Au(111) under driving voltages ranging from 1.4 to 2.5 V. (c) Plot of extracted amplitude value as a function of drive voltage. (d) Comparison of electromechanical response and effective *d*_33, eff_ value in reported monolayer and bilayer 2D materials [32, 35, 56, 57, 58, 59]. (e) Local PFM amplitude and phase hysteresis loops measured with a DC off‐field. (f) PFM phase image showing a box‐in‐box domain pattern written by applying ±4 V bias. (g) False‐color cross‐sectional iDPC‐STEM image of the PFM sample.

Subsequently, we employed dual AC resonance tracking piezoresponse force microscopy (DART‐PFM) to investigate the out‐of‐plane piezoelectric and ferroelectric properties of parallel‐stacked bilayer ReS_2_ on gold. All measurements were conducted directly on the as‐grown samples without transfer (Figure S14a,b), thereby preserving bilayer ReS_2_ intrinsic characteristics. The effective piezoelectric coefficient d_33_ was determined by mapping the PFM amplitude while varying the AC drive voltage from 1.4 to 2.5 V. As shown in Figure 4b, the amplitude contrast of parallel‐stacked bilayer ReS_2_ progressively enhanced with increasing voltage, indicating a strengthened electromechanical response. Linear fitting of the amplitude‐voltage response yields a PFM‐derived apparent electromechanical slope of approximately 3.98 pm V^−1^ for the monolayer ReS_2_/Au(111) region, whereas the parallel‐stacked bilayer region exhibits a substantially larger slope of approximately 9.84 pm V^−1^ (Figure 4c). Although ideal freestanding monolayer ReS_2_ with centrosymmetric P‐1 symmetry cannot exhibit intrinsic piezoelectricity, the supported monolayer ReS_2_/Au(111) heterostructure is asymmetric along the out‐of‐plane direction. Interfacial charge transfer may therefore contribute to its finite interface‐related electromechanical response. The monolayer response provides an experimental reference for interface‐ and measurement‐related contributions and is not assigned as an intrinsic d_33_ of monolayer ReS_2_. In contrast, the larger bilayer response suggests an additional bilayer‐specific electromechanical contribution associated with its polar interlayer registry and is described as a PFM‐derived effective coefficient, d_33, eff_. We note that the two fitted slopes cannot be rigorously subtracted to obtain an intrinsic piezoelectric coefficient because the monolayer and bilayer regions may differ in local electric‐field distribution, contact stiffness, and tip‐sample coupling. Notably, reports on the effective d_33_ of bilayer ReX_2_ (X = S, Se) remain scarce, with a recently reported value of 3.83 pm/V for ∼12‐layer ReSe_2_ being the only available reference [46]. For broader context, we compare electromechanical response of monolayer ReS_2_ and the effective *d*_33, eff_ values of bilayer ReS_2_ with other 2D materials [32, 35, 56, 57, 58, 59] measured via PFM (Figure 4d). The piezoelectric coefficients of parallel‐stacked bilayer ReS_2_ occupy a moderate yet competitive position among 2D systems, comparable to those of CuInP_2_S_6_, underscoring their potential for use in vertical nanopiezoelectric devices.

Local PFM hysteresis measurements further reveal characteristic ferroelectric behavior of the parallel‐stacked bilayer ReS_2_. As shown in Figure 4e, the amplitude response exhibits a characteristic butterfly loop, while the phase response shows a 180° switching under a ±5 V DC bias sweep, with a coercive voltage of approximately ±2 V. To confirm polarization writability, a box‐in‐box pattern was written on the sample by applying ±4 V bias, resulting in a clear phase contrast (Figure 4f), demonstrating reversible polarization switching. These results collectively confirm robust sliding ferroelectricity in parallel‐stacked bilayer ReS_2_. The atomic structure of this sample was later confirmed by FIB‐STEM (Figure S14c) measurement, showing a parallel stacking order configuration (Figure 4g). As an additional control, off‐field PFM hysteresis measurements were performed on monolayer ReS_2_/Au(111) under measurement conditions comparable to those used for the bilayer. The monolayer region shows neither a clear butterfly‐shaped amplitude hysteresis nor an approximately 180° phase reversal (Figure S15), whereas both switching characteristics are observed in the parallel‐stacked bilayer. Because the monolayer control is supported on the same Au(111) substrate and measured under comparable electrostatic and tip‐related conditions, the absence of reversible switching in the monolayer indicates that interfacial charge transfer, electrostatic interactions, and tip‐related effects may contribute to a non‐switchable electromechanical response but are insufficient by themselves to explain the reversible ferroelectric switching observed in the bilayer.

To evaluate possible non‐ferroelectric artifacts, additional conductive AFM (CAFM), off‐resonance PFM, and kelvin probe force microscopy (KPFM) measurements were performed on as‐grown parallel‐stacked bilayer ReS_2_/Au(111) (Figures S16–S18). Local *I–V* curves (Figure S16) show currents below approximately 15 nA within the coercive‐bias window of ±2 V, without abrupt breakdown‐like behavior. Off‐resonance PFM performed at approximately 40 kHz (Figure S17), far below the contact resonance of approximately 350 kHz, still exhibits a phase variation approaching 180° without a corresponding topographic feature. KPFM further reveals two surface‐potential regions differing by approximately 27 mV within a topographically continuous bilayer area (Figure S18), consistent with oppositely polarized domains when considered together with the PFM and DFT results. These control measurements indicate that leakage, electrical breakdown, resonance‐related effects, topographic crosstalk, and static electrostatic interactions are unlikely to be the dominant origin of the observed switching.

To determine whether ferroelectric switching is unique to the parallel‐stacked configuration, we further performed PFM measurements on antiparallel‐stacked bilayer ReS_2_ grown on Au(111) (Figure S19). The antiparallel‐stacked bilayer exhibits a butterfly‐like amplitude hysteresis, an approximately 180° phase reversal, and a box‐in‐box phase pattern, supporting reversible polarization switching. Its coercive bias is approximately 3 V, higher than the approximately 2 V observed for the parallel‐stacked bilayer, which is qualitatively consistent with its larger calculated sliding barrier of approximately 0.17 eV, compared with approximately 0.054 and 0.081 eV for the parallel configuration. Its relatively small PFM amplitude, despite the larger calculated polarization, further indicates that the PFM amplitude reflects the dynamic electromechanical response rather than directly measuring the static polarization magnitude. Although the antiparallel configuration shows a larger calculated polarization and stronger SHG response, the parallel‐stacked bilayer is emphasized for its lower sliding barriers and coercive bias, which favor low‐coercive switching.

Conventional PFM characterization requires transferring materials from their growth substrate or bulk crystal onto conductive substrates (e.g., Pt‐ or Au‐coated substrate) [46, 56]. This process inevitably introduces interfacial contamination and structural damage, potentially modifying the atomic arrangement and obscuring intrinsic ferroelectric properties. To highlight this, we compared the PFM response of our in situ measured CVD grown parallel‐stacked bilayer ReS_2_ on Au(111) with that of transferred CVD bilayer ReS_2_ from Au(111) and exfoliated parallel‐stacked bilayers from bulk ReS_2_. While all three samples exhibited characteristic ferroelectric hysteresis (butterfly amplitude loop and 180° phase switching, Figure 5a,b), quantitative differences are observed. The as‐grown parallel‐stacked bilayer on Au(111) exhibits a maximum PFM amplitude of 400–500 pm, substantially higher than the 250 and 300 pm observed for the transferred and exfoliated parallel‐stacked bilayers, respectively, suggesting a superior piezoelectric response. The coercive voltage also varied markedly: ∼2 V for the as‐grown sample, compared to ∼3 and ∼5 V for the transferred and exfoliated samples. These results demonstrate that the measured ferroelectric response is strongly dependent on the sample configuration and preparation route.

**FIGURE 5:**
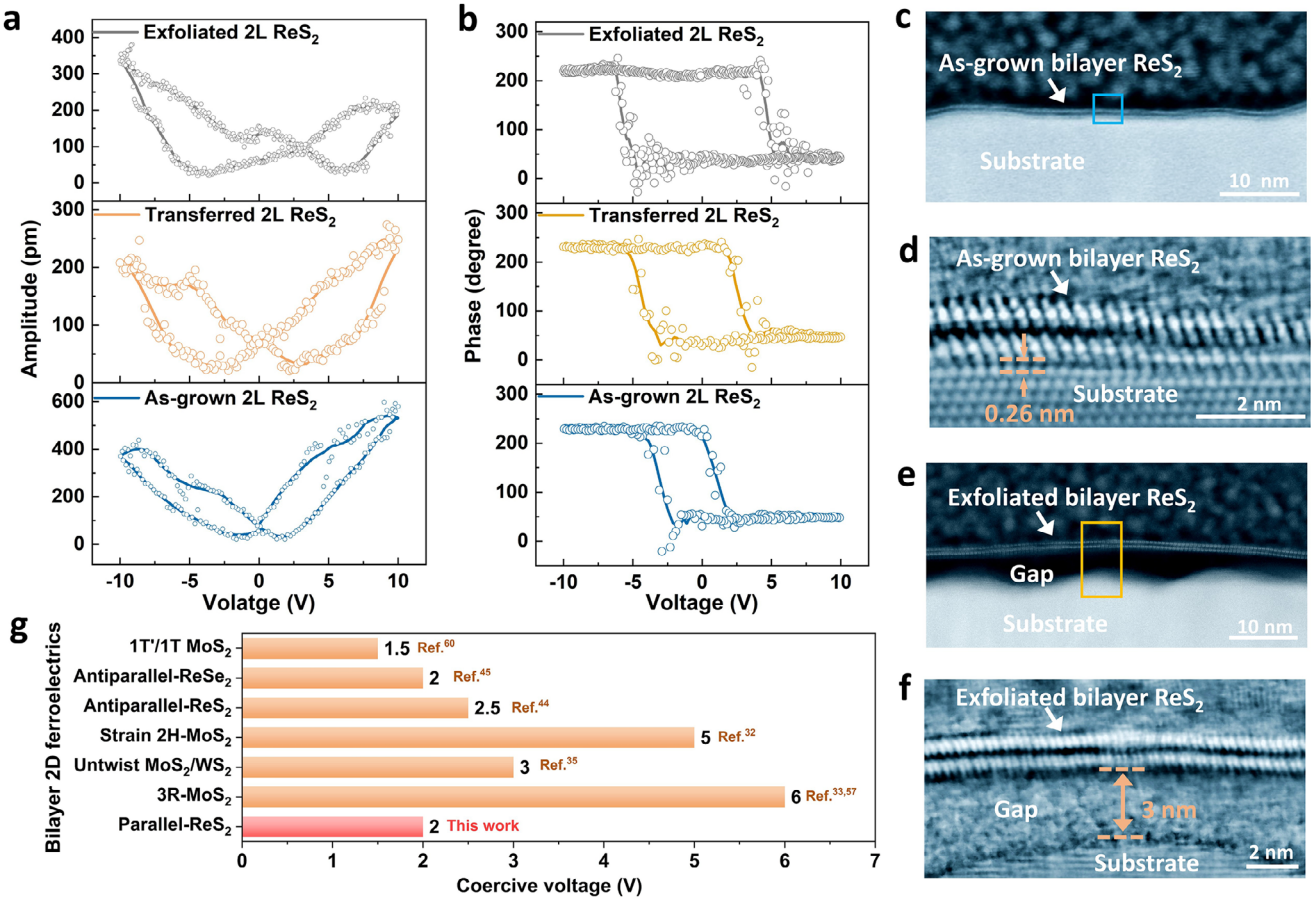
Comparative PFM characterization of parallel‐stacked bilayer ReS_2_ from different preparation methods. (a) PFM amplitude and (b) phase hysteresis loops measured on as‐grown, transferred, and exfoliated parallel‐stacked bilayer (2L) ReS_2_ samples. (c) Low‐magnification HAADF‐STEM image of as‐grown parallel‐stacked bilayer ReS_2_. (d) High‐magnification iDPC‐STEM image of as‐grown parallel‐stacked 2L ReS_2_ from the blue square region marked in (c). (e) Low‐magnification HAADF‐STEM image for exfoliated parallel‐stacked 2L ReS_2_. (f) High‐magnification iDPC‐STEM image of exfoliated parallel‐stacked 2L ReS_2_ from the yellow square region marked in (e). (g) Comparison of PFM coercive voltages in bilayer 2D ferroelectrics [32, 33, 35, 44, 45, 57, 60].

To correlate the above differences with the atomic‐scale structure, we further carried out cross‐sectional STEM analysis on the as‐grown and exfoliated bilayers (Figure 5c–f). The as‐grown CVD bilayer forms a conformal, gap‐free contact that faithfully follows the terraces and steps of the Au(111) substrate (Figure 5c), and the corresponding high‐magnification iDPC‐STEM image (Figure 5d) reveals a well‐ordered bilayer and intimate interfacial contact, characterized by an Au‐S distance of approximately 0.26 nm. By contrast, the exfoliated bilayer ReS_2_, which undergoes a transfer process prior to substrate attachment, displays nanoscale interfacial discontinuities. As shown in Figure 5e, gaps or carbonaceous “dead layers” with dimensions around 2–5 nm (dark contrast regions) are present at the ReS_2_/substrate interface; a locally magnified view is provided in Figure 5f. These features indicate a substantially degraded interfacial quality compared to the as‐grown sample.

Both samples display a parallel stacking order according to local iDPC‐STEM images (Figure 5d,f), indicating that the contrasts in PFM response cannot be attributed to the stacking orientation alone. Such dead‐layer gaps introduce series capacitance and reduce screening, enhancing the depolarization field and diminishing the effective field across ReS_2_. Combined with transfer‐induced strain/defects that pin domains and increase dissipation, this raises the switching barrier and suppresses the piezoelectric displacement. Therefore, the larger PFM response and lower coercive bias observed in the as‐grown sample are consistent with the combined effects of its clean, conformal interface and the absence of transfer‐related artifacts. Direct measurement on the growth substrate thus provides a favorable platform for probing the low‐coercive switching behavior of parallel‐stacked bilayer ReS_2_, although interfacial quality cannot be identified as the sole determining factor.

It is instructive to further compare the switching characteristics of parallel‐stacked bilayer ReS_2_ with those of previously reported bilayer ferroelectrics using a unified experimental metric. Here, we focus on the coercive voltage extracted from PFM measurements, which reflects the local bias required to overcome the polarization switching barrier under highly localized electric‐field conditions. Accordingly, Figure 5g summarizes the reported PFM coercive voltages for representative bilayer ferroelectric systems and compares them with the present results.

In PFM‐based measurements, most bilayer ferroelectric systems exhibit coercive voltages in the range of ∼2–6 V. Within this framework, the parallel‐stacked bilayer ReS_2_ studied here displays a low coercive voltage of ∼2 V, placing it at the lower bound of reported values and below those of bilayer 3R‐MoS_2_ [33, 57], strained 2H‐MoS_2_ [32], and untwisted MoS_2_/WS_2_ heterostructures [35], while being comparable to bilayer antiparallel‐stacked ReX_2_ (X = S, Se) [44, 45] and 1T’/1H MoS_2_ systems [60]. Notably, bilayer 3R‐MoS_2_ requires a comparatively large coercive voltage (∼6 V) in PFM measurements, despite exhibiting a relatively small coercive field (0.05–0.1 V/nm) in device configurations [27, 34]. This contrast underscores that the PFM coercive voltage primarily reflects the local polarization switching barrier rather than macroscopic device‐level coercive field values and cautions against a direct conversion between coercive voltage and coercive field. In this light, the observation of a markedly lower coercive voltage in parallel‐stacked bilayer ReS_2_, despite the unfavorable electrostatic conditions inherent to PFM and the presence of a metallic Au(111) substrate, strongly suggests an intrinsically small polarization switching barrier.

Taken together, the combination of intrinsically low switching barriers, robust bilayer ferroelectricity, and direct growth on metal substrates establishes parallel‐stacked bilayer ReS_2_ as a uniquely promising platform among bilayer two‐dimensional ferroelectrics, particularly for ultra‐low‐power and densely integrated nonvolatile electronic applications.

### 2.5 FTJ and Device‐Level Memory Characteristics of Parallel‐Stacked Bilayer ReS_2_

To evaluate polarization‐dependent electrical transport of parallel‐stacked bilayer ReS_2_, a local tip‐defined conductive AFM tip/parallel‐stacked bilayer ReS_2_/Au(111) ferroelectric tunnel junction (FTJ) was constructed and characterized using CAFM (Figure 6a). During a voltage sweep from −5 to +5 V and subsequently back to −5 V, the junction exhibits a pronounced bipolar hysteretic *I–V* response. Distinct current branches are observed at the same bias depending on the preceding voltage history, consistent with polarization‐dependent switching between high‐ and low‐resistance states. Notably, the current switching occurs on a voltage scale comparable to the PFM coercive biases of approximately ±2 V, linking the polarization‐dependent vertical transport to the low‐coercive switching of parallel‐stacked bilayer ReS_2_. Because the conductive‐state current reaches the approximately ±20 nA limit of the CAFM amplifier, the current switching ratio cannot be accurately extracted from this local FTJ measurement. Moreover, the movable conductive‐tip contact is not suitable for reliable long‐term retention and cycling measurements.

**FIGURE 6:**
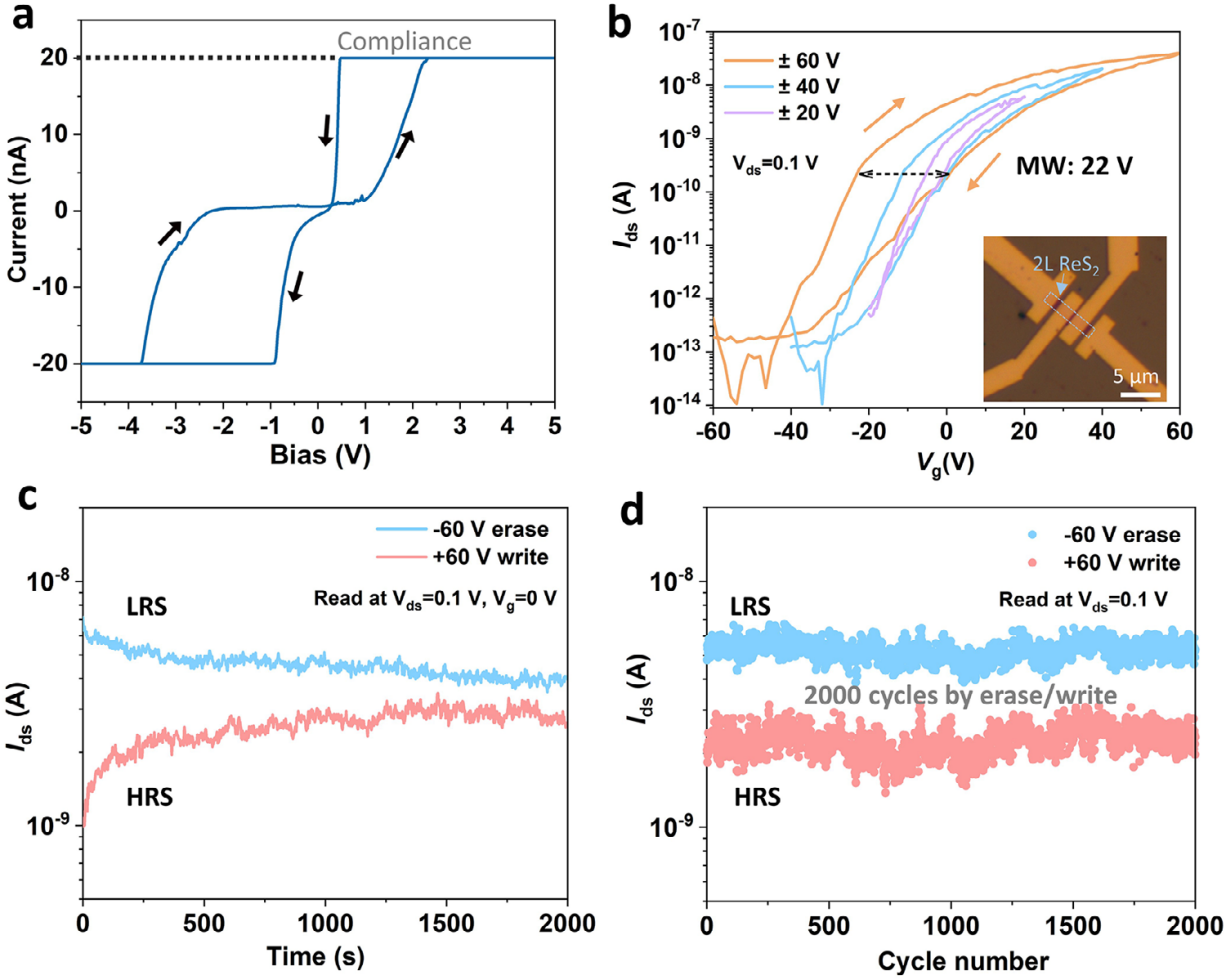
Local ferroelectric tunnel junction and device‐level memory characteristics of parallel‐stacked bilayer ReS_2_. (a) Local *I–V* characteristic of an AFM‐tip/bilayer‐ReS_2_/Au(111) FTJ measured using CAFM. (b) Transfer characteristics of the bilayer ReS_2_ FeFET measured over gate‐voltage ranges of ±20, ±40, and ±60 V. The inset shows an optical image of the device. (c) Retention characteristics of the low‐ and high‐resistance states after −60 V erase and +60 V write operations, respectively. (d) Endurance characteristics of the two resistance states over 2000 erase/write cycles.

To quantitatively evaluate nonvolatile switching under stable device‐level readout conditions, bilayer ReS_2_ ferroelectric field‐effect transistors (FeFET) were further fabricated (Figure 6b–d) by transferring the sample to SiO_2_/Si substrate. The transfer characteristics exhibit sweep‐range‐dependent hysteresis, with the memory window (MW) increasing to approximately 22 V under a ±60 V gate sweep (Figure 6b). The device also exhibits a transistor‐on/off current ratio exceeding 10^5^, demonstrating effective gate modulation of the bilayer ReS_2_ channel. Following −60 V erase and +60 V write operations, the programmed low‐ and high‐resistance states (LRS and HRS) remain distinguishable over 2000 s, with a read‐current ratio of approximately 2 (Figure 6c). The two states also remain reproducibly separated over 2000 erase/write cycles without an obvious collapse of the memory window, maintaining a read‐current ratio on the order of 2 (Figure 6d). These FTJ and FeFET measurements provide complementary electrical evidence for polarization‐dependent vertical transport and reproducible nonvolatile conductance modulation in bilayer ReS_2_.

## 3 Conclusion

In summary, we demonstrate the controlled CVD growth of bilayer ReS_2_ with well‐defined stacking directly on Au(111), establishing a viable route for bilayer low‐symmetry TMDs synthesis on metal substrates. We uncover piezoelectricity and sliding ferroelectricity in parallel‐stacked bilayer ReS_2_ with an ultralow coercive voltage, supported by combined PFM, STEM, and theoretical analysis. FTJ and FeFET measurements further demonstrate nonvolatile electrical switching. Importantly, comparisons among different sample configurations reveal that the cleaner, more conformal interface enabled by direct growth on Au(111) is associated with a lower coercive bias. This work establishes low‐symmetry bilayers as a promising platform for low‐barrier sliding ferroelectrics.

## 4 Experimental Section

### 4.1 Large‐Area Au(111) Preparation and Bilayer ReS_2_ CVD Growth

The Au(111) substrate was prepared by annealing a commercial polycrystal gold foil using the contact‐free annealing method [11]. Bilayer ReS_2_ was synthesized in a dual‐zone CVD furnace. The prepared Au(111) substrate was placed in the center of the downstream zone. A quartz boat containing 10–20 mg of ammonium perrhenate (NH_4_ReO_4_) powder was positioned upstream of the substrate. Separately, 100 mg of sulfur powder in another quartz boat was placed in the upstream zone. Then, the upstream zone of CVD was heated to 250°C within 30 min, while the downstream zone was heated to 800°C–850°C under 150 sccm Ar gas, maintaining growth temperature for 5–10 min. After the heating program concluded, the chamber was allowed to cool naturally to room temperature, resulting in the successful growth of bilayer ReS_2_ on the Au(111) substrate.

### 4.2 Characterization

Sample morphology was characterized using optical microscopy (OM; ZEISS Imager A2m) and scanning electron microscopy (SEM; Helios 5 CX, Thermo Scientific). Raman spectroscopy was performed with a confocal Raman microscope (Renishaw, UK) using 633 nm (for single spectrum and Raman mapping measurement) and 785 nm excitation lasers (for polarized Raman measurements).

Cross‐sectional lamellae of ReS_2_ were prepared by focused ion beam (FIB) milling using a Thermo Scientific Helios 5 CX Dual Beam system. A protective Pt layer (∼200 nm) was first deposited by electron‐beam‐induced deposition, followed by a thicker Pt layer (∼2 µm) deposited by ion‐beam‐induced deposition to minimize ion‐beam damage during milling. Ion‐beam currents of 30 kV at 9.3 and 0.79 nA were used for coarse milling, followed by 80 pA for final thinning. After lamella preparation, surface contamination and ion‐induced damage were further reduced by low‐energy ion polishing at 2 kV and 72 pA.

Atomic‐resolution HAADF‐STEM and iDPC‐STEM images of the ReS_2_, from both top and cross‐sectional views, were acquired using a double spherical aberration‐corrected transmission electron microscope (Thermo Fisher Spectra 300).

Second‐harmonic generation (SHG) measurements were conducted using a high‐resolution, high‐speed multi‐physical scanning system (Metatest, ScanPro Advance) and a WITec alpha300 apyron system with a 1064 nm laser.

Piezoresponse force microscopy (PFM), Kelvin probe force microscopy (KPFM), and conductive AFM (CAFM) measurements were conducted using an Asylum MFP‐3D Infinity and Origin system (Oxford Instruments). PFM was operated in dual AC resonance tracking (DART) mode and single‐frequency PFM mode using Pt/Ir‐coated Si cantilevers. DART‐PFM imaging was performed with an AC voltage of 1–3 V at a contact‐resonance frequency of approximately 350 kHz, whereas off‐resonance PFM imaging was conducted at approximately 40 kHz. Local piezoresponse hysteresis loops were acquired using on/off‐field switching spectroscopy with DC bias voltages of ±5 or ±10 V. The off‐field hysteresis loops were analyzed to evaluate ferroelectric switching behavior while minimizing electrostatic contributions. Ferroelectric domain writing was carried out in lithography‐PFM mode using DC voltages of ±4 V, followed by readout under an AC bias of 1–2 V.

KPFM was directly performed on as‐grown bilayer ReS_2_/Au(111) samples using Pt/Ir‐coated cantilevers to map the local surface‐potential distribution. KPFM measurements were conducted using a dual‐pass lift‐mode procedure. During the first pass, the surface topography was acquired in tapping mode at a resonance frequency of approximately 75 kHz under a drive amplitude ∼1 V. During the second pass, the tip retraced the recorded topographic profile at a fixed lift height (0–30 nm), while a feedback‐controlled DC bias was applied to compensate for the contact potential difference between the conductive tip and the sample. The compensating voltage was recorded as the local surface potential.

CAFM measurements were performed directly on the as‐grown ReS_2_/Au(111) samples using Pt/Ir‐coated conductive tip and a dedicated optimized resistance conductance amplifier (ORCA) conductive cantilever holder. The sample was mounted on the sample stage while remaining electrically isolated from ground. A bias wire from the ORCA holder was connected directly to the Au substrate to apply the sample bias, while the conductive tip was maintained at virtual ground. Local *I–V* curves were acquired at multiple positions using a bias sweep within −5 to +5 V. For the local tip‐defined ferroelectric tunnel junction measurements, a complete sample‐bias sweep from −5 to +5 V and subsequently back to −5 V was applied.

### 4.3 Transfer of Bilayer ReS_2_

The bilayer ReS_2_/Au sample was spin‐coated with a layer of PMMA A4. The coated sample was then immersed in a gold etchant (KI–I_2_ solution) until the gold substrate was completely etched away. The floating PMMA/ReS_2_ film was subsequently rinsed three times in deionized (DI) water to remove any residual etchant. Finally, the film was transferred onto the target substrate, such as a TEM grid, a Pt‐coated Si substrate, and SiO_2_/Si substrate, and the PMMA support layer was dissolved by immersion in acetone.

### 4.4 Device Fabrication and Measurement

The parallel‐stacked bilayer ReS_2_ was first transferred to a 300 nm SiO_2_/Si substrate. Electrodes were patterned by electron‐beam lithography using a TESCAN MIRA system, followed by the deposition of Cr/Au electrodes (10/50 nm) using a Denton Explorer electron‐beam evaporation system. The ferroelectric field‐effect transistors (FeFETs) were tested by the semiconductor analyzer (Keysight B1500A) in a probe station (Lake Shore PS‐100) under vacuum and dark environment. Transfer characteristics were measured at a drain‐source voltage of *V*_ds_ = 0.1 V using gate‐voltage sweep ranges of ±20, ±40, and ±60 V. For retention measurements, the device was programmed using gate voltages of −60 and +60 V, corresponding to the low‐ and high‐resistance states, respectively, and the drain current was subsequently monitored at *V*_g_ = 0 V and *V*_ds_ = 0.1 V. Endurance characteristics were evaluated by repeatedly applying −60 and +60 V erase/write operations for 2000 cycles, with the programmed states read at *V*_g_ = 0 V and *V*_ds_ = 0.1 V.

### 4.5 DFT Calculations

All first‐principles calculations were carried out using the Vienna Ab initio Simulation Package (VASP.6.3.2) with the core‐electron interactions described by the projector wave‐augmented method [61, 62]. The Perdew–Burke–Ernzerhof parametrized generalized gradient approximation (PBE‐GGA) was used for the exchange‐correlation interaction [63]. The total energy and structural relaxation convergence criteria were set to 1 × 10^−6^ eV and 0.01 eV/Å, respectively. In addition, the DFT‐D3 method with the Becke–Johnson damping function was included to account for the weak van der Waals interactions [64, 65]. The kinetic energy cutoff of the plane‐wave basis was chosen to be 400 eV. The Brillouin zones were sampled using a *k*‐point mesh of 5 × 5 × 1 respectively. The polarization (*p*) of 2D materials were calculated by following equation, $p=\frac{1}{A}\;\int r\rho dr^{3}$, where *A* and ρ are the areas of in‐plane ReS_2_ and charge density, respectively, and r is the position of charge.

## Author Contributions

**Yuhuan Li**: investigation, data curation. **Tianren Chen**: investigation. **Honglin Chen**: investigation, writing – original draft. **Jiong Zhao**: investigation, writing – review and editing, supervision. **Ke Yang**: investigation. **Ming Yang**: funding acquisition, supervision, writing – review and editing. **Lingli Huang**: investigation. **Thuc Hue Ly**: conceptualization, investigation, funding acquisition, methodology, validation, writing – review and editing, formal analysis, supervision, resources, project administration.

## Conflicts of Interest

The authors declare no conflicts of interest.

## Supporting information




**Supporting File**: adma74537‐sup‐0001‐SuppMat.docx.

## Data Availability

The data that support the findings of this study are available from the corresponding author upon reasonable request.
