# Supplementary material for: Direct Growth of 2D Bilayers on Au(111) for Low‐Coercive Sliding Ferroelectricity
Source: Adv Mater. 2026 Aug 5;38(56):e74537. doi: 10.1002/adma.74537 (PMC13648928; doi:10.1002/adma.74537)
Supplement: Supplementary file 1 [file ADMA-38-e74537-s001.docx]

Supporting Information

**Direct Growth of 2D Bilayers on Au (111) for Low-Coercive Sliding Ferroelectricity**

*Honglin Chen,^1,2,+^ Ke Yang,^2,+^ Yuhuan Li,^2^ Lingli Huang,^1,3^ Tianren Chen,^2^* *Ming Yang,^2,8,9,^* Jiong Zhao,^2,4,7,^* and Thuc Hue Ly^1,3,5,6,^**

*^1^* Department of Chemistry and Center of Super-Diamond & Advanced Films, City University of Hong Kong, Kowloon, Hong Kong SAR, China.

^2^ Department of Physics and Materials, The Hong Kong Polytechnic University, Kowloon, Hong Kong SAR, China.

^3^ City University of Hong Kong Shenzhen Research Institute, Shenzhen, China.

^4^ The Hong Kong Polytechnic University Shenzhen Research Institute, Shenzhen, China.

*^5^* Department of Chemistry and State Key Laboratory of Marine Environmental Health, City University of Hong Kong, Kowloon, Hong Kong SAR, China.

*^6^* Hong Kong Institute for Clean Energy, City University of Hong Kong, Kowloon, Hong Kong SAR, China.

^7^ The Research Institute for Advanced Manufacturing, The Hong Kong Polytechnic University, Kowloon, Hong Kong SAR, China.

^8^ Research Centre on Data Sciences & Artificial Intelligence, The Hong Kong Polytechnic University, Kowloon, Hong Kong SAR, China.

^9^ Research Centre for Nanoscience and Nanotechnology, The Hong Kong Polytechnic University, Kowloon, Hong Kong SAR, China.

^+^These authors contributed equally.

*Corresponding authors: kevin.m.yang@polyu.edu.hk (Ming Yang); jiong.zhao@polyu.edu.hk (Jiong Zhao); thuchly@cityu.edu.hk (Thuc Hue Ly).

**Figure S1.** Re source-dependent growth experiment under CVD configuration in Figure1c, i. (a) Photography image for used Au (111) substrate. (b) OM image for ReS_2_ grown gold with gradually increased Re precursors.

**Figure S2.** Tuning ReS_2_ growth via Re precursor-gold distance and growth temperature. (a-e) OM images showing the growth result for (a-c) Re precursor-gold distance-dependent and (c-e) growth temperature-dependent experiments. (f) Raman spectrum of the by-products formed in the condition shown in (c).

**Figure S3.** The influence of substrate height on ReS_2_ growth. (a) Schematic and OM image of ReS_2_ synthesized at substrate height h=2 cm. (b) Schematic and OM image of ReS_2_ synthesized at substrate height h=0 cm.

**Figure S4.** Growth evolution of ReS_2_ from monolayer (1L) flakes to a continuous bilayer (2L) film. (a-f) OM and SEM images of ReS_2_ growth evolution with the increase of Re precursors.

Figure S5. Large-area Raman mapping of an as-grown bilayer ReS2 film on Au (111). (a) Optical image of the mapped region. (b) Raman intensity map of the characteristic mode at 213 cm-1 over a 50 × 50 μm^2^ area. (c) Representative Raman spectrum extracted from the bilayer region in (b), together with a reference spectrum acquired from the monolayer region shown in (d). (d) Optical microscopy image of a partially covered bilayer ReS2 region, showing the optical contrast between the monolayer and bilayer areas for reference.

**Figure S6.** The investigation of H_2_ flow rate on bilayer (2L) ReS_2_ growth. (a-c) OM and SEM images of ReS_2_ synthesized under different H_2_ flow rates. (d) Evolution of monolayer, bilayer, and multilayer coverage as a function of H_2_ flow rate.

**Figure S7.** Angle-resolved polarized Raman characterization and structural models of bilayer ReS_2_ on Au (111). (a) Angle-resolved polarized Raman intensity polar plots for characteristic peaks III and V. (b) Top-view atomic models illustrating the two distinct stacking configurations: $\bar{\text{c}}$-up (c-axis comes into the plane) and c-up (c-axis comes out the plane) structure.

**Figure S8.** Top-view STEM characterization and SAED analysis of bilayer ReS_2_. (a-f) Representative HAADF-STEM images showing diverse stacking configurations of bilayer ReS_2_ with distinct Re atomic patterns arising from interlayer sliding. (g-i) TEM images and corresponding SAED patterns of three representative bilayer ReS_2_ samples, each showing a single set of diffraction spots indicative of identical lattice orientations in the two layers.

**Figure S9.** False-color iDPC-STEM images of two types of stacking orders. (a-c) False-color iDPC-STEM image of parallel-stacked bilayer ReS_2_. (d-f) False-color iDPC-STEM image of antiparallel-stacked bilayer ReS_2_.

**Figure S10.** OM image, SHG spectrum and cross-sectional STEM images used to confirm the stacking configurations of bilayer samples exhibiting strong and weak SHG signals. (a) OM image of three selected bilayer samples transferred from Au (111) onto SiO_2_/Si. The blue square indicates the FIB cutting region. (b) SHG signal collected from the three samples shown in (a). (c) Low- and high-magnification cross-sectional STEM images of the three samples in (a). The results confirm that samples 1 and 3 with weak SHG signals correspond to parallel stacking, while sample 2 with a strong SHG signal corresponds to antiparallel stacking.

**Figure S11.** Statistical analysis of nucleation modes and stacking configurations for bilayer ReS_2_ on Au (111). Low- and high-magnification cross-sectional STEM images of bilayer ReS_2_ grown via (a) layer-by-layer, (b) homo-site, and (c) hetero-site nucleation modes, together with their corresponding stacking configurations. Among the 11 identified stacking configurations (a1-a7, b1, b2, c1 and c2), 6 are parallel-stacked.

**Figure S12.** Theoretical analysis of ferroelectricity in antiparallel-stacked bilayer ReS_2_. (a) Top- and side-view atomic models of antiparallel-stacked bilayer ReS_2_. (b) The energy map of bilayer ReS_2_ as a function of sliding distance along a and b axis. Two ferroelectric states (polar states A’ and B’) and the intermediate state C’ are indicated by red dots. (c) The side-view atomic models of polar states A’ and B’ and the corresponding iDPC-STEM images. (d) The energy pathway for the ferroelectric transition from polar state A’ to B’. (e) The charge density difference of bilayer ReS_2_ in polar state A’. The purple and blue regions represent charge accumulation and depletion, respectively.

**Figure S13.** Log-log plot of SHG intensity as a function of excitation power.

**Figure S14.** The bilayer ReS_2_/Au(111) used for both PFM and FIB-STEM study. (a) OM image of ReS_2_/Au(111) where PFM measurements were conducted. (b) Corresponding AFM topography image of the area shown in (a) and Figure 4b. (c) Low-magnification HAADF-STEM image of a FIB-prepared cross-section cut from the rectangle region outlined in (a).

**Figure S15.** Local PFM amplitude and phase hysteresis loops measured on monolayer area with a DC off-field.

**Figure S16.** Local *I-V* curves acquired on as-grown parallel-stacked bilayer ReS_2_ on Au (111).

**Figure S17.** Off-resonance single-frequency PFM measurement of as-grown parallel-stacked bilayer ReS_2_ on Au (111). (a) Topography and (b) PFM phase image acquired at a fixed excitation frequency of approximately 40 kHz. (c) Phase profile along the line indicated in panel (b), showing a phase variation approaching 180°. (d) DART-PFM phase image for comparison.

**Figure S18.** KPFM characterization of as-grown parallel-stacked bilayer ReS_2_ on Au (111). (a) Topography, (b) surface-potential map, and (c) potential profile along the dashed arrow in panel (b).

**Figure S19.** PFM measurement of antiparallel-stacked ReS_2_ on Au (111). (a) OM image of antiparallel-stacked bilayer ReS2 on Au (111). (b) Local PFM amplitude and phase hysteresis loops measured with a DC off-field. (c) PFM phase image showing a box-in-box domain pattern written by applying ±4 V bias. (d) False-color cross-sectional iDPC-STEM image of the PFM sample.
